# Two new polytypes of 2,4,6-tri­bromo­benzo­nitrile

**DOI:** 10.1107/S2056989016000256

**Published:** 2016-01-13

**Authors:** Doyle Britton, Wayland E. Noland, Kenneth J. Tritch

**Affiliations:** a Department of Chemistry, University of Minnesota, Minneapolis, MN 55455-0431, USA

**Keywords:** crystal structure, polytypes, polymorphs, Sandmeyer, isocyanide, N⋯Br contacts, C⋯Br contacts

## Abstract

Three polymorphs of 2,4,6-tri­bromo­benzo­nitrile (RCN), C_7_H_2_Br_3_N, two of which are novel and one of which is a redetermination of the original structure first determined by Carter & Britton [(1972). *Acta Cryst.* B**28**, 945–950] are found to be polytypic. Each has a layer structure which differs only in the stacking of the layers. Each layer is composed of mol­ecules associated through C≡N⋯Br contacts which form *R* ^2^_2_(10) rings. Two such rings are associated with each N atom; one with each *ortho*-Br atom. No new polytypes of 1,3,5-tri­bromo-2-iso­cyano­benzene (RNC) were found but a re-determination of the original structure by Carter *et al.* [(1977). *Cryst. Struct. Commun.* **6**, 543–548] is presented. RNC was found to be isostructural with one of the novel polytypes of RCN. Unit cells were determined for 23 RCN samples and 11 RNC samples. Polytypes could not be distinguished based on crystal habits. In all four structures, each mol­ecule of the asymmetric unit lies across a mirror plane.

## Abstract 2

Two new polymorphs of 2,4,6-tri­bromo­benzo­nitrile have been found. Together with the known polymorph, they are polytypic. One new polytype is isostructural with the previously reported crystal structure of 1,3,5-tri­bromo-2-iso­cyano­benzene.

## Chemical context   

**Figure 1:**
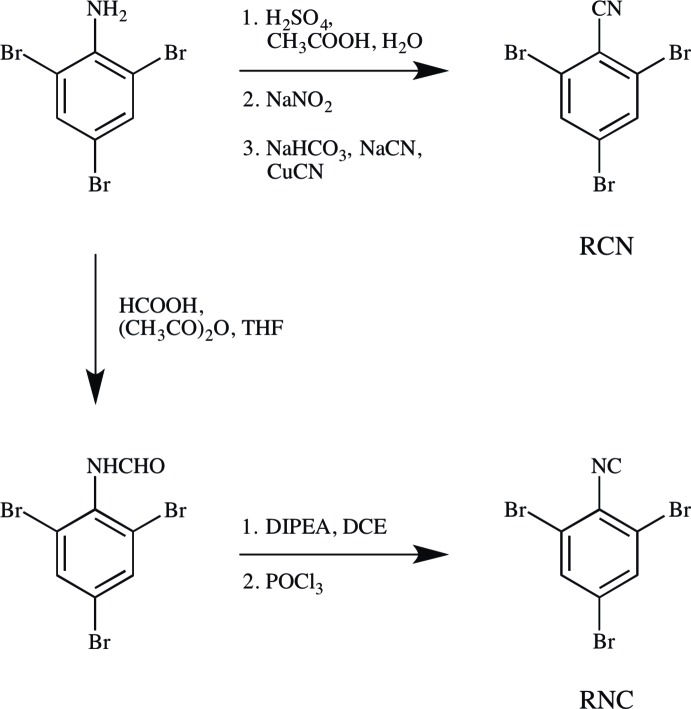
Synthesis of RCN and RNC.

The reported structures of 2,4,6-tri­bromo­benzo­nitrile (RCN, Figs. 1 ▸ and 2 ▸; Carter & Britton, 1972 ▸) and 1,3,5-tri­bromo-2-iso­cyano­benzene (RNC, Figs. 1 ▸ and 3 ▸; Carter *et al.*, 1977 ▸) have two-dimensional layers of similarly arranged mol­ecules, but the packing of adjacent layers is distinctly different. At the time, no explanation was offered. It was puzzling, given that the two compounds are isoelectronic, isosteric, and the principal inter­molecular inter­actions, C≡N⋯Br and N≡C⋯Br, are similar. Recent reports of polytype organic structures, such as picryl bromide (Parrish *et al.*, 2008 ▸) and 5,6-di­methyl­benzofurazan 1-oxide (Britton *et al.*, 2012 ▸) led to the idea that RCN and RNC might occur as polytypes. Earlier, Bredig (1930 ▸) had determined the space group and unit cell of RCN with the same results as Carter & Britton. Bredig was trying to follow up on the goniometer studies of Jaeger (1909 ▸), but while he found the same *a*:*b* ratio as Jaeger in the RCN unit cell, he found a different *b*:*c* ratio.
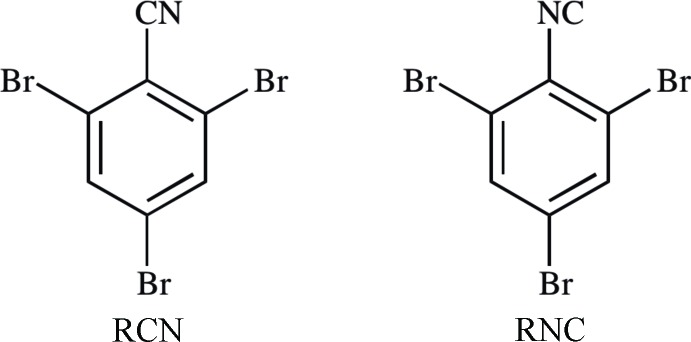


Accordingly, a search was made for polytypes of RCN, and to a lesser extent, of RNC. Four different structures were identified. RCN-I is the original *Z* = 2 structure of RCN; RCN-II is a new *Z* = 8 polytype; RCN-III is a new *Z* = 12 polytype. No RNC counterparts to RCN-I or RCN-III were observed. RNC-II is the original *Z* = 8 structure. As the *Z* values suggest, RCN-II and RNC-II are isomorphs.

## Structural commentary   

Mol­ecules of RCN and RNC are nearly planar. The average distance of atoms from the plane of best fit is 0.025 Å in RCN-I. For RCN-II, the average distances are 0.037 and 0.010 Å, for the (N27) and (N37) mol­ecules, respectively. In RNC-II, the mol­ecules are slightly more distorted, with average deviations of 0.043 and 0.017 Å for the (N127) and (N137) mol­ecules, respectively. For RCN-III, the average distances are 0.009, 0.018, and 0.032 Å for the (N47), (N57), and (N67) mol­ecules, respectively.

**Figure 4:**
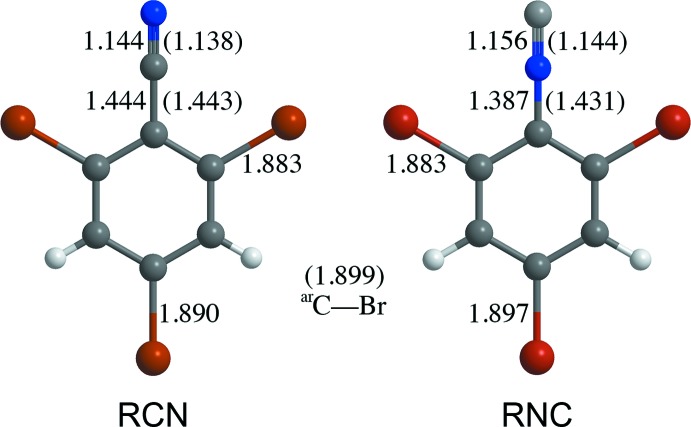
Selected bond lengths (Å) in RCN and RNC, averaged across all polytypes. The data shown in parentheses are the mean distances for each bond type reported by Allen *et al.* (1987 ▸).

The bond lengths in RCN and RNC are generally similar (Fig. 4 ▸). They are also similar to the mean bond distances reported for bonds of each type (Allen *et al.*, 1987 ▸). The N atom in RNC is displaced toward the aryl ring compared to the literature distances for aryl isocyanides.

## Supra­molecular features   

**Figure 5:**
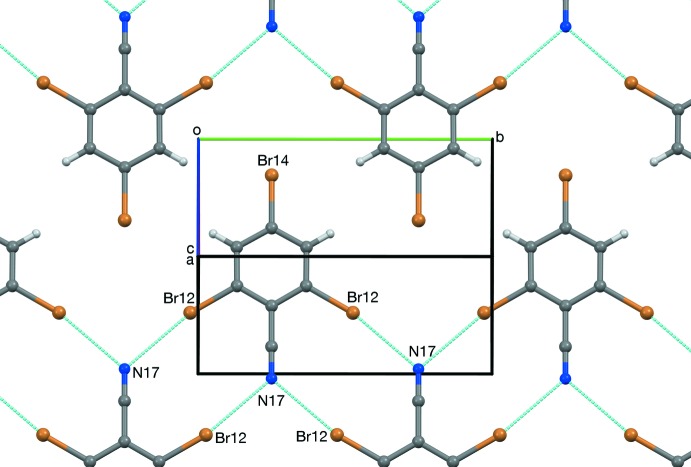
View of one layer of RCN-I along [10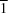]. Dashed blue lines represent short contacts.

**Figure 6:**
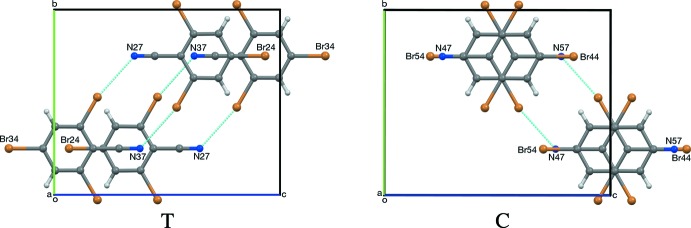
Pseudotranslational (T) and pseudocentric (C) stacking of layers in RCN-II and RCN-III, respectively. Both are viewed along [100]. The mol­ecules shown are the second pair of layers from the top, in Fig. 7 ▸ and Fig. 8 ▸, respectively.

**Figure 7:**
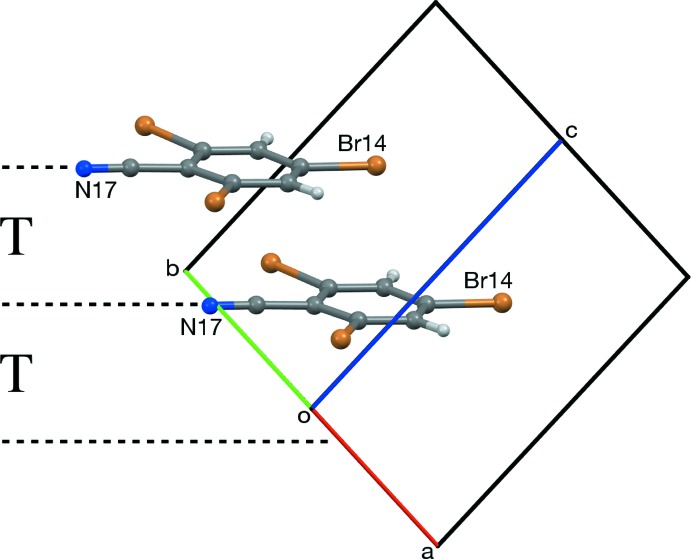
Translational (T) stacking of layers in *Z* = 2 RCN-I, viewed along [110]. If the unit cell of RCN-I is transformed by the matrix [100/010/201], the dimensions of the projection become 10.247 (3) × 12.480 (3) Å, which is similar to the corresponding *b* × *c* measurements, 10.2147 (10) × 12.4754 (12) Å for RCN-II, and 10.2167 (18) × 12.493 (2) Å for RCN-III.

**Figure 8:**
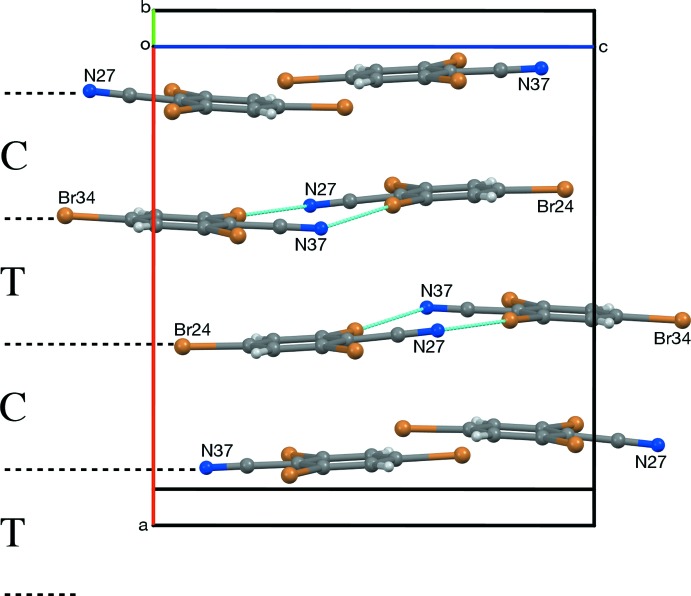
Pseudocentric (C) and pseudotranslational (T) stacking of layers in *Z* = 8 RCN-II, viewed roughly along [010].

**Figure 9:**
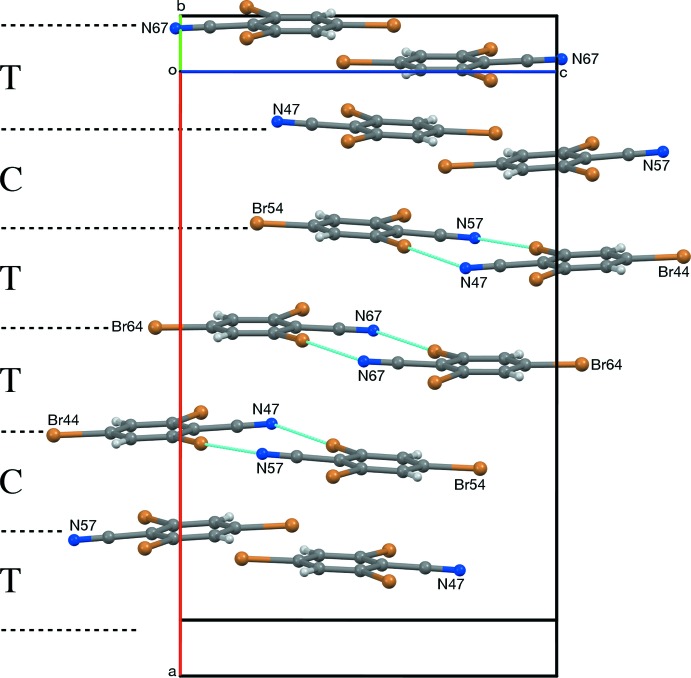
Pseudotranslational (T) and pseudocentric (C) stacking of layers in *Z* = 12 RCN-III, viewed roughly along [010].

Fig. 5 ▸ shows a two-dimensional layer of RCN-I. All of the structures are composed of similar layers. Adjacent mol­ecules are associated through C≡N⋯Br inter­actions, arranged in 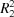
(10) rings (Etter, 1990 ▸; Bernstein *et al.*, 1995 ▸). The CN⋯Br distances in these rings range between 3.053 and 3.077 Å (Table 1 ▸); these distances can be compared with the N⋯Br van der Waals distance of 3.40 Å (Bondi, 1964 ▸; Rowland & Taylor, 1996 ▸). Each layer in RCN-II is composed of alternating (N27) and (N37) mol­ecules. RCN-III contains two layers of alternating (N47) and (N57) mol­ecules for each layer composed entirely of (N67) mol­ecules. Adjacent pairs of layers show translational or pseudotranslational, or pseudocentric stacking (Fig. 6 ▸). RCN-I shows translational stacking between all adjacent layers (Fig. 7 ▸). In RCN-II, alternating pairs of layers show pseudocentric and pseudotranslational stacking (Fig. 8 ▸). In RCN-III, each layer of (N67) mol­ecules pseudotranslationally overlaps both neighboring (N47/N57) layers, while pairs of adjacent (N47/N57) layers, every third pair of layers, overlap pseudocentrically (Fig. 9 ▸).

The NC⋯Br contact distances in RNC-II are a smaller percentage of the van der Waals distance, 3.63 Å, *versus* corresponding atoms in RCN-II. The contacts in RNC-II occur at slightly wider angles than those in RCN-II (Table 1 ▸).

In RCN-II, the planes of best fit of the two different mol­ecules are inclined by 6.5° to each other; in RNC-II this inclination is 7.5°. In RCN-III, the relative inclination of planes of (N47) and (N57) mol­ecules is 7.0°. These two planes are approximately bis­ected by the planes of (N67) mol­ecules.

## Database survey   

A search of the Cambridge Structural Database (Version 5.36, update 3; Groom & Allen, 2014 ▸) for 2,4,6-trihalo-3,5-unsubstituted benzo­nitriles found nine entries: RCN; its tri­chloro analog, Gol’der *et al.* (1952 ▸), Carter & Britton (1972 ▸), Pink *et al.* (2000 ▸); its tri­fluoro analog, Britton (2008 ▸); four mixed-halogen entries, Gleason & Britton (1978 ▸), Britton (2005 ▸), Britton *et al.* (2002 ▸), and Britton (1997 ▸). Searching for the corresponding isocyanides found two entries: RNC and its tri­chloro analog (Pink *et al.*, 2000 ▸).

Layers of the type observed in RCN were reported in 2,6-di­bromo entries with Cl, Br, or I at the 4-position. Other entries exhibit short contacts between the cyano- or iso­cyano- group and one *ortho*-halogen atom of an intra­layer mol­ecule, with various inter­layer contacts. Polymorphs are only reported for 2,4,6-tri­chloro­benzo­nitrile; those are not polytypic.

Expanding the search to include organometallic complexes found three more entries, with the cyano N or iso­cyano C atom ligating gallium (tri­fluoro­benzo­nitrile; Tang *et al.*, 2012 ▸), rhenium (tri­chloro­iso­cyano­benzene; Ko *et al.*, 2011 ▸), and ruthenium (RNC; Leung *et al.*, 2009 ▸).

## Synthesis and crystallization   

**2,4,6-Tri­bromo­aniline** was prepared from aniline according to the work of Coleman & Talbot (1943 ▸).

**RCN**, adapted from the work of Toya *et al.* (1992 ▸): *Diazo­tization:* 2,4,6-Tri­bromo­aniline (1.25 g), water (2.5 ml), and glacial acetic acid (4.4 ml) were combined in a round-bottomed flask. The resulting suspension was cooled in an ice bath, and then H_2_SO_4_ (98%, 1.0 ml) was added dropwise, followed by an ice-cold solution of NaNO_2_ (520 mg) in water (4 ml). The resulting mixture was warmed to 310 K for 1 h, and then cooled in an ice bath. *Cyanide suspension:* CuCN (680 mg) and NaCN (1.12 g) were dissolved in water (20 ml). NaHCO_3_ (10.9 g) and ethyl acetate (10 ml) were added, giving a suspension, which was cooled in an ice bath. *Cyanation:* The diazo­tization mixture was added dropwise to the cyanide suspension as quickly as possible without causing excessive foaming. The ice bath was removed and then the mixture was stirred overnight. The organic phase was set aside. The aqueous phase was extracted with ethyl acetate (3 × 10 ml). The combined organic portions were washed with brine (10 ml), dried with Na_2_SO_4_, and concentrated at reduced pressure, giving a brown powder, which was purified by column chromatography (SiO_2_, hexa­ne–ethyl acetate, gradient from 1:0 to 10:1). The desired fraction (*R_f_* = 0.61 in 8:1) was concentrated at reduced pressure, giving beige needles (760 mg, 59%). M.p. 400–400.5 K (lit. 402 K; Giumanini *et al.*, 1996 ▸); ^1^H NMR (300 MHz, CD_2_Cl_2_) δ 7.853 (*s*, H13); ^13^C NMR (75 MHz, CD_2_Cl_2_) δ 135.3 (C13), 128.6 (C14), 127.4 (C12), 118.3 (C17), 116.0 (C11); IR (NaCl, cm^−1^) 3095, 3068, 2921 (*w*), 2233 (*s*, C≡N; lit. 2232), 1716 (*w*), 1563 (*s*), 1527 (*s*), 1431 (*s*), 1410 (*s*), 1370 (*s*), 1353 (*s*), 1328, 1191 (*s*), 1109 (*s*), 1087, 1063 (*s*), 854 (*s*), 809 (*s*), 748 (*s*); MS (EI, *m/z*) [*M*]^+^ calculated for C_7_H_2_Br_3_N 336.7732, found 336.7716.

**2,4,6-Tri­bromo­formanilide**, adapted from the work of Krishnamurthy (1982 ▸): Acetic anhydride (3.2 ml) and tetra­hydro­furan (THF, 5.0 ml) were combined in a round-bottomed flask. Formic acid (88% aq., 1.7 ml) was added dropwise. The resulting solution was stirred for 30 min at room temperature. A solution of 2,4,6-tri­bromo­aniline (1.82 g) in THF (20 ml) was added dropwise. The resulting mixture was stirred for 18 h. The resulting heterogeneous mixture was filtered through neutral alumina (Sigma–Aldrich 199974, 5 cm H × 3 cm D), with addition of sufficient THF to elute all product, as indicated by TLC. The filtrate was concentrated at reduced pressure. The resulting residue was washed with sat. NaHCO_3_ solution (50 ml), and then filtered. The filter cake was recrystallized from acetone, giving white needles (1.72 g, 87%). M.p. 493–494 K (lit. 494.5 K; Chattaway *et al.*, 1899 ▸); *R_f_* = 0.48 (SiO_2_ in 1:1 hexa­ne–ethyl acetate); ^1^H NMR (300 MHz, (CD_3_)_2_SO) δ 10.192 (*s*, N*H*, *O-E* conformer, 0.87H), 8.522 (*s*, N*H*, *O-Z* conformer, 0.13H), 8.260 (*s*, C*H*O, 1H), 8.018 (*s*, C*H*, 2H); ^13^C NMR (75 MHz, (CD_3_)_2_SO) δ 165.9 (*C*O, *O-Z* conformer), 159.8 (*C*O, *O-E* conformer), 134.6 (*ipso*-*C*), 134.4 (*C*H), 124.5 (*ortho*-*C*Br), 121.1 (*para*-*C*Br); IR (NaCl, cm^−1^) 3201, 3166, 1661 (*s*, C=O), 1558, 1154, 858, 810; MS (ESI, *m/z*) [*M* – H]^−^ calculated for C_7_H_4_Br_3_NO 355.7750, found 355.7758. Analysis (MHW Laboratories, Phoenix, AZ, USA) calculated for C_7_H_4_Br_3_NO: C 23.50, H 1.13, Br 66.99, N 3.91; found C 23.42, H 1.15, Br 66.71, N 3.57.

**RNC**, adapted from the work of Ugi *et al.* (1965 ▸): 2,4,6-Tri­bromo­formanilide (1.96 g) and *N*,*N*-diiso­propyl­ethyl­amine (DIPEA, 3.4 ml) were added to 1,2-di­chloro­ethane (75 ml). The resulting suspension was refluxed for 5 min, and then cooled to room temperature. POCl_3_ (0.6 ml) was added dropwise. The mixture was stirred for 18 h, cooled in an ice bath, and then filtered through neutral alumina (3 cm H × 3 cm D), with addition of sufficient di­chloro­methane (DCM) to elute all product as indicated by TLC. The filtrate was concentrated at reduced pressure. The resulting yellow residue was dissolved in DCM (25 ml), cooled in an ice bath, and washed with ice-cold acetic acid solution (0.025 M, 3 × 15 ml), and then ice-cold sat. NaHCO_3_ solution (15 ml). The organic phase was collected, dried with Na_2_SO_4_, and then concentrated under a stream of nitro­gen, giving beige needles upon filtration (630 mg, 34%). M.p. 390 K (lit. 394 K, Mironov & Mokrushin, 1999 ▸); *R_f_* = 0.75 (Al_2_O_3_ in 2:1 hexa­ne–ethyl acetate); ^1^H NMR (300 MHz, CD_2_Cl_2_) δ 7.827 (*s*, H123); ^13^C NMR (75 MHz, (CD_3_)_2_CO) 159.7 (C127), 135.8 (C123), 135.4 (C121), 124.5 (C124), 122.0 (C122); IR (NaCl, cm^−1^) 3162, 3068, 2921, 2128 (*s*, N≡C; lit. 2125), 1660 (*s*), 1555 (*s*), 1370 (*s*), 856 (*s*), 701 (*s*); MS (EI, *m/z*) [*M*]^+^ calculated for C_7_H_2_Br_3_N 336.7732, found 336.7734.

**Crystallization:** RCN crystals were grown by slow evaporation of single-solvent solutions (290–295 K). RCN-I was obtained from aceto­nitrile, benzene, chloro­form, or methyl­ene chloride; RCN-II from mesitylene; RCN-III from benzene or chloro­form. RNC-II crystals were obtained by sublimation (385 K, 0.05 torr), or by slow evaporation from the same solvents as RCN (268–295 K).

## Refinement   

Crystal data, data collection, and structure refinement details for RCN and RNC are summarized in Table 2 ▸. H atoms were placed in calculated positions and refined as riding atoms, with C—H = 0.95 Å and *U*_iso_(H) = 1.2*U*_eq_(C).

## Supplementary Material

Crystal structure: contains datablock(s) global, RCN-I, RCN-II, RCN-III, RNC-II. DOI: 10.1107/S2056989016000256/lh5796sup1.cif

Structure factors: contains datablock(s) RCN-I. DOI: 10.1107/S2056989016000256/lh5796RCN-Isup2.hkl

Supporting information file. DOI: 10.1107/S2056989016000256/lh5796RCN-Isup6.cml

Structure factors: contains datablock(s) RCN-II. DOI: 10.1107/S2056989016000256/lh5796RCN-IIsup3.hkl

Structure factors: contains datablock(s) RCN-III. DOI: 10.1107/S2056989016000256/lh5796RCN-IIIsup4.hkl

Structure factors: contains datablock(s) RNC-II. DOI: 10.1107/S2056989016000256/lh5796RNC-IIsup5.hkl

CCDC references: 1445499, 1445498, 1445497, 1445496

Additional supporting information:  crystallographic information; 3D view; checkCIF report

## Figures and Tables

**Figure 2:**
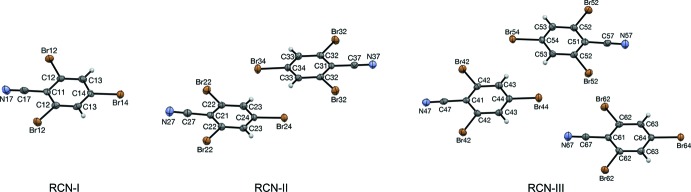
Mol­ecular structures, with atom labeling, of RCN-I viewed along [11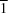]; RCN-II viewed along [120]; RCN-III viewed along [120]. Displacement ellipsoids are drawn at the 50% probability level. In discussion, mol­ecules are named by their respective nitro­gen atoms. Each mol­ecule lies across a crystallographic mirror plane.

**Figure 3:**
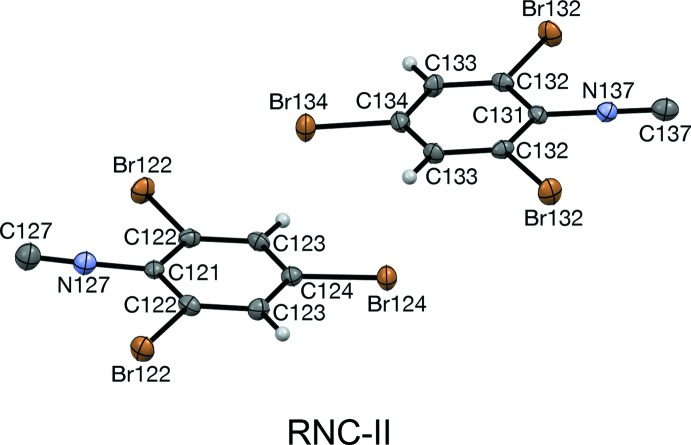
Mol­ecular structure, with atom labeling, of RNC-II viewed along [120]. Displacement ellipsoids are drawn at the 50% probability level. Each mol­ecule lies across a crystallographic mirror plane.

**Table 1:** Short contact geometry (Å, °)

*X*≡*Y*⋯Br	*X*≡*Y*	*Y*⋯Br	*X*≡*Y*⋯Br
C17≡N17⋯Br12^i^	1.144 (10)	3.053 (4)	131.45 (9)
C27≡N27⋯Br32^ii^	1.132 (7)	3.059 (3)	131.76 (7)
N127≡C127⋯Br132^ii^	1.147 (6)	3.141 (4)	134.01 (8)
C37≡N37⋯Br22^iii^	1.156 (6)	3.077 (3)	130.68 (10)
N137≡C137⋯Br122^iii^	1.164 (6)	3.161 (4)	133.23 (11)
C47≡N47⋯Br52^ii^	1.146 (6)	3.072 (3)	130.95 (9)
C57≡N57⋯Br42^iii^	1.147 (6)	3.057 (3)	131.47 (7)
C67≡N67⋯Br62^iv^	1.139 (6)	3.065 (3)	131.96 (7)

Symmetry codes: (i) −*x*, 1 − *y*, −*z*; (ii) *x*, *y*, −1 + *z*; (iii) *x*, *y*, 1 + *z*; (iv) 1 − *x*, 1 − *y*, 1 − *z*.

**Table 2:** Experimental details

	RCN-I	RCN-II	RCN-III	RNC-II
Crystal data				
Chemical formula	C_7_H_2_Br_3_N	C_7_H_2_Br_3_N	C_7_H_2_Br_3_N	C_7_H_2_Br_3_N
*M*_r_	339.83	339.83	339.83	339.83
Crystal system, space group	Monoclinic, *P*2_1_/*m*	Orthorhombic, *P* *n* *m* *a*	Orthorhombic, *P* *n* *m* *a*	Orthorhombic, *P* *n* *m* *a*
Temperature (K)	173	173	173	173
*a*, *b*, *c* (Å)	4.8742 (15), 10.247 (3), 8.683 (3)	13.6183 (13), 10.2147 (10), 12.4754 (12)	20.399 (4), 10.2167 (18), 12.493 (2)	13.5916 (18), 10.1464 (13), 12.6158 (16)
α, β, γ (°)	90, 94.97 (1), 90	90, 90, 90	90, 90, 90	90, 90, 90
*V* (Å^3^)	432.0 (2)	1735.4 (3)	2603.7 (8)	1739.8 (4)
*Z*	2	8	12	8
Radiation type	Mo *K*α	Mo *K*α	Mo *K*α	Mo *K*α
μ (mm^−1^)	13.93	13.88	13.87	13.84
Crystal size (mm)	0.50 × 0.15 × 0.10	0.25 × 0.20 × 0.07	0.50 × 0.15 × 0.10	0.40 × 0.35 × 0.20

Data collection				
Diffractometer	Bruker 1K area detector	Bruker 1K area detector	Bruker 1K area detector	Bruker APEXII CCD
Absorption correction	Multi-scan (*SADABS*; Bruker, 2002 ▸)	Multi-scan (*SADABS*; Bruker, 2002 ▸)	Multi-scan (*SADABS*; Bruker, 2002 ▸)	Multi-scan (*SADABS*; Bruker, 2002 ▸)
*T*_min_, *T*_max_	0.080, 0.248	0.06, 0.37	0.054, 0.337	0.170, 0.333
No. of measured, independent and observed [*I* > 2σ(*I*)] reflections	4093, 1024, 856	16607, 2093, 1692	22804, 2691, 2165	19459, 2105, 1638
*R*_int_	0.127	0.052	0.055	0.078
(sin θ/λ)_max_ (Å^−1^)	0.649	0.650	0.616	0.650

Refinement				
*R*[*F* ^2^ > 2σ(*F* ^2^)], *wR*(*F* ^2^), *S*	0.046, 0.116, 1.01	0.028, 0.063, 1.02	0.023, 0.046, 1.07	0.025, 0.055, 1.06
No. of reflections	1024	2093	2691	2105
No. of parameters	58	115	173	116
H-atom treatment	H-atom parameters constrained	H-atom parameters constrained	H-atom parameters constrained	H-atom parameters constrained
Δρ_max_, Δρ_min_ (e Å^−3^)	1.36, −1.28	0.44, −0.69	0.56, −0.49	0.44, −0.48

Computer programs: *SMART*, *APEX2* and *SAINT* (Bruker, 2002 ▸), *SHELXT* (Sheldrick, 2015*a* ▸), *SHELXL2014* (Sheldrick, 2015*b* ▸), *Mercury* (Macrae *et al.*, 2008 ▸), *SHELXTL* (Sheldrick, 2008 ▸), *enCIFer* (Allen *et al.*, 2004 ▸), and *publCIF* (Westrip, 2010 ▸).

## supplementary crystallographic information

### (RCN-I) 2,4,6-Tribromobenzonitrile - polytype I Crystal data

**Table:**

C_7_H_2_Br_3_N	*F*(000) = 312
*M**_r_* = 339.83	*D*_x_ = 2.612 Mg m^−^^3^
Monoclinic, *P*2_1_/*m*	Mo *K*α radiation, λ = 0.71073 Å
Hall symbol: -P 2yb	Cell parameters from 2049 reflections
*a* = 4.8742 (15) Å	θ = 2.4–27.4°
*b* = 10.247 (3) Å	µ = 13.93 mm^−^^1^
*c* = 8.683 (3) Å	*T* = 173 K
β = 94.97 (1)°	Needle, colorless
*V* = 432.0 (2) Å^3^	0.50 × 0.15 × 0.10 mm
*Z* = 2	

### (RCN-I) 2,4,6-Tribromobenzonitrile - polytype I Data collection

**Table:**

Bruker 1K area-detector diffractometer	1024 independent reflections
Radiation source: fine-focus sealed tube	856 reflections with *I* > 2σ(*I*)
Graphite monochromator	*R*_int_ = 0.127
ω scans	θ_max_ = 27.5°, θ_min_ = 2.4°
Absorption correction: multi-scan (*SADABS*; Bruker, 2002)	*h* = −6→6
*T*_min_ = 0.080, *T*_max_ = 0.248	*k* = −13→13
4093 measured reflections	*l* = −11→11

### (RCN-I) 2,4,6-Tribromobenzonitrile - polytype I Refinement

**Table:**

Refinement on *F*^2^	Primary atom site location: structure-invariant direct methods
Least-squares matrix: full	Secondary atom site location: difference Fourier map
*R*[*F*^2^ > 2σ(*F*^2^)] = 0.046	Hydrogen site location: inferred from neighbouring sites
*wR*(*F*^2^) = 0.116	H-atom parameters constrained
*S* = 1.01	*w* = 1/[σ^2^(*F*_o_^2^) + (0.069*P*)^2^] where *P* = (*F*_o_^2^ + 2*F*_c_^2^)/3
1024 reflections	(Δ/σ)_max_ = 0.001
58 parameters	Δρ_max_ = 1.36 e Å^−^^3^
0 restraints	Δρ_min_ = −1.28 e Å^−^^3^

### (RCN-I) 2,4,6-Tribromobenzonitrile - polytype I Fractional atomic coordinates and isotropic or equivalent isotropic displacement parameters (Å^2^)

**Table:**

	*x*	*y*	*z*	*U*_iso_*/*U*_eq_	
Br12	0.33356 (11)	0.47324 (5)	0.18676 (7)	0.0280 (2)	
Br14	1.11323 (14)	0.7500	0.57820 (9)	0.0256 (3)	
N17	−0.0263 (14)	0.7500	−0.0147 (8)	0.0313 (16)	
C11	0.3828 (14)	0.7500	0.1960 (8)	0.0204 (15)	
C12	0.4932 (10)	0.6324 (5)	0.2559 (6)	0.0224 (11)	
C13	0.7107 (10)	0.6313 (5)	0.3688 (6)	0.0244 (11)	
H13	0.7842	0.5512	0.4091	0.029*	
C14	0.8200 (14)	0.7500	0.4224 (8)	0.0197 (15)	
C17	0.1523 (16)	0.7500	0.0799 (9)	0.0241 (16)	

### (RCN-I) 2,4,6-Tribromobenzonitrile - polytype I Atomic displacement parameters (Å^2^)

**Table:**

	*U*^11^	*U*^22^	*U*^33^	*U*^12^	*U*^13^	*U*^23^
Br12	0.0334 (4)	0.0160 (3)	0.0337 (4)	−0.0043 (2)	−0.0015 (2)	−0.0016 (2)
Br14	0.0229 (4)	0.0239 (4)	0.0295 (5)	0.000	−0.0018 (3)	0.000
N17	0.041 (4)	0.021 (3)	0.031 (4)	0.000	−0.006 (3)	0.000
C11	0.022 (3)	0.025 (4)	0.015 (4)	0.000	0.005 (3)	0.000
C12	0.023 (2)	0.016 (2)	0.029 (3)	−0.0012 (19)	0.006 (2)	0.001 (2)
C13	0.024 (2)	0.017 (3)	0.033 (3)	0.004 (2)	0.007 (2)	0.004 (2)
C14	0.024 (3)	0.025 (4)	0.011 (3)	0.000	0.003 (3)	0.000
C17	0.030 (4)	0.011 (3)	0.032 (4)	0.000	0.004 (3)	0.000

### (RCN-I) 2,4,6-Tribromobenzonitrile - polytype I Geometric parameters (Å, º)

**Table:**

Br12—C12	1.883 (5)	C12—C13	1.380 (8)
Br14—C14	1.881 (7)	C13—C14	1.391 (6)
C11—C12	1.401 (6)	C13—H13	0.9500
C11—C17	1.443 (10)	N17—C17	1.144 (10)
			
C12—C11—C12^i^	118.6 (6)	C12—C13—H13	120.7
C12—C11—C17	120.7 (3)	C14—C13—H13	120.7
C13—C12—C11	121.2 (5)	C13—C14—C13^i^	121.9 (6)
C13—C12—Br12	119.3 (4)	C13—C14—Br14	119.0 (3)
C11—C12—Br12	119.4 (4)	N17—C17—C11	178.4 (9)
C12—C13—C14	118.6 (5)		
			
C12^i^—C11—C12—C13	−1.6 (11)	C11—C12—C13—C14	−0.2 (10)
C17—C11—C12—C13	−178.9 (7)	Br12—C12—C13—C14	−177.8 (5)
C12^i^—C11—C12—Br12	176.0 (3)	C12—C13—C14—C13^i^	2.0 (12)
C17—C11—C12—Br12	−1.3 (9)	C12—C13—C14—Br14	179.2 (5)

Symmetry code: (i) *x*, −*y*+3/2, *z*.

### (RCN-II) 2,4,6-Tribromobenzonitrile - polytype II Crystal data

**Table:**

C_7_H_2_Br_3_N	*F*(000) = 1248
*M**_r_* = 339.83	*D*_x_ = 2.601 Mg m^−^^3^
Orthorhombic, *P**n**m**a*	Mo *K*α radiation, λ = 0.71073 Å
Hall symbol: -P 2ac 2n	Cell parameters from 3180 reflections
*a* = 13.6183 (13) Å	θ = 2.9–27.2°
*b* = 10.2147 (10) Å	µ = 13.88 mm^−^^1^
*c* = 12.4754 (12) Å	*T* = 173 K
*V* = 1735.4 (3) Å^3^	Plate, colorless
*Z* = 8	0.25 × 0.20 × 0.07 mm

### (RCN-II) 2,4,6-Tribromobenzonitrile - polytype II Data collection

**Table:**

Bruker 1K area-detector diffractometer	2093 independent reflections
Radiation source: fine-focus sealed tube	1692 reflections with *I* > 2σ(*I*)
Graphite monochromator	*R*_int_ = 0.052
ω scans	θ_max_ = 27.5°, θ_min_ = 2.2°
Absorption correction: multi-scan (*SADABS*; Bruker, 2002)	*h* = −17→17
*T*_min_ = 0.06, *T*_max_ = 0.37	*k* = −13→13
16607 measured reflections	*l* = −16→16

### (RCN-II) 2,4,6-Tribromobenzonitrile - polytype II Refinement

**Table:**

Refinement on *F*^2^	Primary atom site location: structure-invariant direct methods
Least-squares matrix: full	Secondary atom site location: difference Fourier map
*R*[*F*^2^ > 2σ(*F*^2^)] = 0.028	Hydrogen site location: inferred from neighbouring sites
*wR*(*F*^2^) = 0.063	H-atom parameters constrained
*S* = 1.02	*w* = 1/[σ^2^(*F*_o_^2^) + (0.030*P*)^2^ + 1.560*P*] where *P* = (*F*_o_^2^ + 2*F*_c_^2^)/3
2093 reflections	(Δ/σ)_max_ = 0.001
115 parameters	Δρ_max_ = 0.44 e Å^−^^3^
0 restraints	Δρ_min_ = −0.69 e Å^−^^3^

### (RCN-II) 2,4,6-Tribromobenzonitrile - polytype II Fractional atomic coordinates and isotropic or equivalent isotropic displacement parameters (Å^2^)

**Table:**

	*x*	*y*	*z*	*U*_iso_*/*U*_eq_	
Br22	0.13341 (3)	0.52761 (3)	0.04244 (3)	0.02658 (11)	
Br24	0.14558 (4)	0.2500	0.43375 (4)	0.02477 (13)	
C21	0.1318 (3)	0.2500	0.0608 (4)	0.0197 (10)	
C22	0.1359 (2)	0.3683 (3)	0.1174 (3)	0.0206 (7)	
C23	0.1418 (2)	0.3697 (3)	0.2282 (3)	0.0217 (7)	
H23	0.1444	0.4500	0.2666	0.026*	
C24	0.1437 (3)	0.2500	0.2821 (4)	0.0190 (10)	
C27	0.1207 (4)	0.2500	−0.0545 (4)	0.0257 (11)	
N27	0.1115 (3)	0.2500	−0.1447 (4)	0.0332 (11)	
Br32	0.10699 (3)	0.47273 (3)	0.69146 (3)	0.02650 (11)	
Br34	0.12804 (4)	0.7500	0.29979 (4)	0.02786 (13)	
C31	0.1095 (3)	0.7500	0.6720 (3)	0.0175 (9)	
C32	0.1116 (2)	0.6320 (3)	0.6155 (3)	0.0195 (7)	
C33	0.1171 (2)	0.6315 (3)	0.5049 (3)	0.0201 (7)	
H33	0.1189	0.5511	0.4666	0.024*	
C34	0.1199 (3)	0.7500	0.4508 (4)	0.0196 (10)	
C37	0.1056 (3)	0.7500	0.7873 (4)	0.0200 (10)	
N37	0.1015 (3)	0.7500	0.8798 (3)	0.0255 (9)	

### (RCN-II) 2,4,6-Tribromobenzonitrile - polytype II Atomic displacement parameters (Å^2^)

**Table:**

	*U*^11^	*U*^22^	*U*^33^	*U*^12^	*U*^13^	*U*^23^
Br22	0.0364 (2)	0.01742 (19)	0.0259 (2)	−0.00058 (15)	−0.00196 (15)	0.00451 (14)
Br24	0.0303 (3)	0.0261 (3)	0.0179 (2)	0.000	0.00098 (19)	0.000
C21	0.018 (2)	0.021 (3)	0.021 (2)	0.000	0.0017 (19)	0.000
C22	0.0208 (16)	0.0182 (16)	0.0227 (17)	0.0016 (14)	−0.0003 (13)	0.0017 (14)
C23	0.0225 (16)	0.0186 (18)	0.0240 (17)	−0.0012 (14)	−0.0012 (14)	−0.0017 (14)
C24	0.021 (2)	0.021 (3)	0.015 (2)	0.000	0.0022 (18)	0.000
C27	0.028 (3)	0.020 (3)	0.029 (3)	0.000	0.000 (2)	0.000
N27	0.048 (3)	0.026 (2)	0.026 (3)	0.000	−0.002 (2)	0.000
Br32	0.0386 (2)	0.01618 (19)	0.02475 (19)	−0.00117 (15)	0.00205 (14)	0.00374 (14)
Br34	0.0418 (3)	0.0243 (3)	0.0174 (2)	0.000	−0.0003 (2)	0.000
C31	0.017 (2)	0.021 (2)	0.015 (2)	0.000	−0.0003 (17)	0.000
C32	0.0182 (15)	0.0163 (16)	0.0241 (17)	0.0004 (13)	−0.0006 (13)	0.0044 (14)
C33	0.0229 (17)	0.0157 (18)	0.0216 (17)	0.0015 (14)	−0.0009 (13)	−0.0018 (14)
C34	0.025 (2)	0.018 (2)	0.015 (2)	0.000	−0.0001 (18)	0.000
C37	0.023 (2)	0.014 (2)	0.023 (3)	0.000	−0.0009 (19)	0.000
N37	0.030 (2)	0.024 (2)	0.023 (2)	0.000	−0.0002 (17)	0.000

### (RCN-II) 2,4,6-Tribromobenzonitrile - polytype II Geometric parameters (Å, º)

**Table:**

Br22—C22	1.877 (3)	Br32—C32	1.884 (3)
Br24—C24	1.892 (5)	Br34—C34	1.887 (4)
C21—C22	1.400 (4)	C31—C32	1.396 (4)
C21—C27	1.446 (7)	C31—C37	1.439 (6)
C22—C23	1.385 (5)	C32—C33	1.382 (5)
C23—C24	1.395 (4)	C33—C34	1.387 (4)
C23—H23	0.9500	C33—H33	0.9500
C27—N27	1.132 (7)	C37—N37	1.156 (6)
			
C22^i^—C21—C22	119.3 (4)	C32^ii^—C31—C32	119.3 (4)
C22—C21—C27	120.3 (2)	C32—C31—C37	120.3 (2)
C23—C22—C21	120.9 (3)	C33—C32—C31	120.6 (3)
C23—C22—Br22	119.3 (3)	C33—C32—Br32	120.0 (3)
C21—C22—Br22	119.8 (3)	C31—C32—Br32	119.4 (2)
C22—C23—C24	118.2 (3)	C32—C33—C34	118.9 (3)
C22—C23—H23	120.9	C32—C33—H33	120.5
C24—C23—H23	120.9	C34—C33—H33	120.5
C23^i^—C24—C23	122.4 (4)	C33^ii^—C34—C33	121.6 (4)
C23—C24—Br24	118.8 (2)	C33—C34—Br34	119.2 (2)
N27—C27—C21	179.7 (5)	N37—C37—C31	179.3 (5)
			
C22^i^—C21—C22—C23	−1.4 (6)	C32^ii^—C31—C32—C33	0.8 (6)
C27—C21—C22—C23	176.8 (4)	C37—C31—C32—C33	−178.9 (4)
C22^i^—C21—C22—Br22	178.6 (2)	C32^ii^—C31—C32—Br32	−179.2 (2)
C27—C21—C22—Br22	−3.2 (5)	C37—C31—C32—Br32	1.1 (5)
C21—C22—C23—C24	0.1 (5)	C31—C32—C33—C34	−0.3 (5)
Br22—C22—C23—C24	−179.9 (3)	Br32—C32—C33—C34	179.7 (3)
C22—C23—C24—C23^i^	1.3 (7)	C32—C33—C34—C33^ii^	−0.2 (7)
C22—C23—C24—Br24	−177.1 (2)	C32—C33—C34—Br34	179.7 (2)

Symmetry codes: (i) *x*, −*y*+1/2, *z*; (ii) *x*, −*y*+3/2, *z*.

### (RCN-III) 2,4,6-Tribromobenzonitrile - polytype III Crystal data

**Table:**

C_7_H_2_Br_3_N	*F*(000) = 1872
*M**_r_* = 339.83	*D*_x_ = 2.601 Mg m^−^^3^
Orthorhombic, *P**n**m**a*	Mo *K*α radiation, λ = 0.71073 Å
Hall symbol: -P 2ac 2n	Cell parameters from 2928 reflections
*a* = 20.399 (4) Å	θ = 2.6–26.7°
*b* = 10.2167 (18) Å	µ = 13.87 mm^−^^1^
*c* = 12.493 (2) Å	*T* = 173 K
*V* = 2603.7 (8) Å^3^	Needle, colorless
*Z* = 12	0.50 × 0.15 × 0.10 mm

### (RCN-III) 2,4,6-Tribromobenzonitrile - polytype III Data collection

**Table:**

Bruker 1K area-detector diffractometer	2691 independent reflections
Radiation source: fine-focus sealed tube	2165 reflections with *I* > 2σ(*I*)
Graphite monochromator	*R*_int_ = 0.055
ω scans	θ_max_ = 26.0°, θ_min_ = 2.6°
Absorption correction: multi-scan (*SADABS*; Bruker, 2002)	*h* = −24→24
*T*_min_ = 0.054, *T*_max_ = 0.337	*k* = −12→12
22804 measured reflections	*l* = −15→15

### (RCN-III) 2,4,6-Tribromobenzonitrile - polytype III Refinement

**Table:**

Refinement on *F*^2^	Secondary atom site location: difference Fourier map
Least-squares matrix: full	Hydrogen site location: inferred from neighbouring sites
*R*[*F*^2^ > 2σ(*F*^2^)] = 0.023	H-atom parameters constrained
*wR*(*F*^2^) = 0.046	*w* = 1/[σ^2^(*F*_o_^2^) + (0.0096*P*)^2^ + 3.390*P*] where *P* = (*F*_o_^2^ + 2*F*_c_^2^)/3
*S* = 1.07	(Δ/σ)_max_ = 0.001
2691 reflections	Δρ_max_ = 0.56 e Å^−^^3^
173 parameters	Δρ_min_ = −0.49 e Å^−^^3^
0 restraints	Extinction correction: *SHELXL*, Fc^*^=kFc[1+0.001xFc^2^λ^3^/sin(2θ)]^-1/4^
Primary atom site location: structure-invariant direct methods	Extinction coefficient: 0.00028 (3)

### (RCN-III) 2,4,6-Tribromobenzonitrile - polytype III Fractional atomic coordinates and isotropic or equivalent isotropic displacement parameters (Å^2^)

**Table:**

	*x*	*y*	*z*	*U*_iso_*/*U*_eq_	
Br42	0.340999 (16)	0.52705 (3)	−0.05464 (3)	0.02707 (10)	
Br44	0.32895 (2)	0.2500	0.33679 (4)	0.02913 (13)	
Br52	0.332551 (16)	0.47245 (3)	0.59427 (3)	0.02775 (9)	
Br54	0.32011 (2)	0.7500	0.20370 (3)	0.02542 (12)	
Br62	0.511839 (16)	0.52774 (3)	0.67598 (3)	0.02743 (10)	
Br64	0.50730 (2)	0.2500	1.06666 (4)	0.02435 (12)	
C41	0.33919 (19)	0.2500	−0.0353 (3)	0.0182 (9)	
C42	0.33772 (14)	0.3675 (3)	0.0214 (2)	0.0207 (7)	
C43	0.33432 (14)	0.3686 (3)	0.1321 (2)	0.0226 (7)	
H43	0.3331	0.4487	0.1706	0.027*	
C44	0.3328 (2)	0.2500	0.1851 (4)	0.0219 (10)	
C47	0.3440 (2)	0.2500	−0.1508 (4)	0.0218 (10)	
N47	0.34814 (18)	0.2500	−0.2423 (3)	0.0272 (9)	
C51	0.3338 (2)	0.7500	0.5758 (4)	0.0221 (10)	
C52	0.33096 (14)	0.6320 (3)	0.5193 (2)	0.0211 (7)	
C53	0.32641 (14)	0.6314 (3)	0.4085 (2)	0.0228 (7)	
H53	0.3246	0.5512	0.3701	0.027*	
C54	0.3245 (2)	0.7500	0.3549 (3)	0.0204 (10)	
C57	0.3399 (2)	0.7500	0.6908 (4)	0.0225 (10)	
N57	0.3445 (2)	0.7500	0.7823 (3)	0.0329 (10)	
C61	0.5080 (2)	0.2500	0.6942 (4)	0.0204 (10)	
C62	0.50889 (14)	0.3676 (3)	0.7509 (2)	0.0216 (7)	
C63	0.50886 (14)	0.3686 (3)	0.8618 (2)	0.0218 (7)	
H63	0.5092	0.4488	0.9002	0.026*	
C64	0.5083 (2)	0.2500	0.9155 (4)	0.0200 (10)	
C67	0.5049 (2)	0.2500	0.5783 (4)	0.0225 (10)	
N67	0.5024 (2)	0.2500	0.4872 (3)	0.0329 (10)	

### (RCN-III) 2,4,6-Tribromobenzonitrile - polytype III Atomic displacement parameters (Å^2^)

**Table:**

	*U*^11^	*U*^22^	*U*^33^	*U*^12^	*U*^13^	*U*^23^
Br42	0.03864 (19)	0.01711 (17)	0.02545 (18)	−0.00178 (14)	0.00143 (15)	0.00391 (15)
Br44	0.0432 (3)	0.0252 (3)	0.0190 (3)	0.000	0.0007 (2)	0.000
Br52	0.03826 (19)	0.01818 (17)	0.02682 (18)	−0.00040 (15)	−0.00267 (15)	0.00467 (15)
Br54	0.0309 (3)	0.0270 (3)	0.0184 (2)	0.000	0.0017 (2)	0.000
Br62	0.03877 (19)	0.01736 (17)	0.02617 (18)	−0.00080 (15)	−0.00036 (15)	0.00376 (15)
Br64	0.0300 (2)	0.0243 (3)	0.0188 (2)	0.000	−0.00018 (19)	0.000
C41	0.016 (2)	0.017 (2)	0.022 (2)	0.000	−0.0010 (18)	0.000
C42	0.0214 (15)	0.0175 (17)	0.0233 (17)	−0.0001 (14)	−0.0011 (13)	0.0052 (14)
C43	0.0269 (16)	0.0160 (17)	0.0248 (17)	−0.0016 (14)	−0.0003 (14)	−0.0016 (15)
C44	0.023 (2)	0.024 (3)	0.019 (2)	0.000	0.0000 (19)	0.000
C47	0.020 (2)	0.016 (2)	0.029 (3)	0.000	−0.004 (2)	0.000
N47	0.033 (2)	0.022 (2)	0.026 (2)	0.000	−0.0017 (19)	0.000
C51	0.016 (2)	0.026 (3)	0.024 (2)	0.000	0.001 (2)	0.000
C52	0.0244 (15)	0.0154 (17)	0.0234 (16)	0.0002 (14)	−0.0002 (13)	0.0037 (14)
C53	0.0255 (16)	0.0198 (18)	0.0232 (17)	0.0025 (14)	0.0024 (14)	−0.0031 (15)
C54	0.020 (2)	0.024 (3)	0.017 (2)	0.000	0.0021 (18)	0.000
C57	0.025 (2)	0.015 (2)	0.027 (3)	0.000	−0.003 (2)	0.000
N57	0.048 (3)	0.027 (2)	0.024 (2)	0.000	−0.001 (2)	0.000
C61	0.020 (2)	0.022 (2)	0.020 (2)	0.000	0.0041 (19)	0.000
C62	0.0199 (15)	0.0188 (17)	0.0261 (17)	0.0017 (13)	0.0011 (13)	0.0047 (15)
C63	0.0236 (16)	0.0178 (18)	0.0239 (16)	0.0007 (14)	0.0018 (13)	−0.0028 (15)
C64	0.020 (2)	0.022 (2)	0.018 (2)	0.000	0.0017 (19)	0.000
C67	0.028 (2)	0.016 (2)	0.024 (3)	0.000	0.000 (2)	0.000
N67	0.055 (3)	0.021 (2)	0.024 (2)	0.000	−0.002 (2)	0.000

### (RCN-III) 2,4,6-Tribromobenzonitrile - polytype III Geometric parameters (Å, º)

**Table:**

Br42—C42	1.888 (3)	C51—C52	1.399 (4)
Br44—C44	1.897 (5)	C51—C57	1.443 (6)
Br52—C52	1.880 (3)	C52—C53	1.387 (4)
Br54—C54	1.892 (4)	C53—C54	1.384 (4)
Br62—C62	1.885 (3)	C53—H53	0.9500
Br64—C64	1.889 (4)	C57—N57	1.147 (6)
C41—C42	1.394 (4)	C61—C62	1.395 (4)
C41—C47	1.447 (6)	C61—C67	1.450 (6)
C42—C43	1.384 (4)	C62—C63	1.386 (4)
C43—C44	1.381 (4)	C63—C64	1.385 (4)
C43—H43	0.9500	C63—H63	0.9500
C47—N47	1.146 (6)	C67—N67	1.139 (6)
			
C42—C41—C42^i^	118.9 (4)	C54—C53—H53	120.6
C42—C41—C47	120.5 (2)	C52—C53—H53	120.6
C43—C42—C41	121.0 (3)	C53^ii^—C54—C53	122.1 (4)
C43—C42—Br42	119.9 (2)	C53—C54—Br54	119.0 (2)
C41—C42—Br42	119.2 (2)	N57—C57—C51	179.8 (5)
C44—C43—C42	118.3 (3)	C62^i^—C61—C62	119.0 (4)
C44—C43—H43	120.9	C62—C61—C67	120.5 (2)
C42—C43—H43	120.9	C63—C62—C61	120.9 (3)
C43^i^—C44—C43	122.6 (4)	C63—C62—Br62	119.4 (3)
C43—C44—Br44	118.7 (2)	C61—C62—Br62	119.8 (2)
N47—C47—C41	179.7 (5)	C64—C63—C62	118.6 (3)
C52^ii^—C51—C52	119.1 (4)	C64—C63—H63	120.7
C52—C51—C57	120.4 (2)	C62—C63—H63	120.7
C53—C52—C51	120.7 (3)	C63^i^—C64—C63	122.0 (4)
C53—C52—Br52	119.7 (2)	C63—C64—Br64	119.0 (2)
C51—C52—Br52	119.7 (2)	N67—C67—C61	180.0 (5)
C54—C53—C52	118.7 (3)		
			
C42^i^—C41—C42—C43	−0.5 (6)	C51—C52—C53—C54	−0.2 (5)
C47—C41—C42—C43	−178.8 (3)	Br52—C52—C53—C54	179.2 (3)
C42^i^—C41—C42—Br42	179.07 (19)	C52—C53—C54—C53^ii^	−0.6 (6)
C47—C41—C42—Br42	0.8 (5)	C52—C53—C54—Br54	178.7 (2)
C41—C42—C43—C44	0.4 (5)	C62^i^—C61—C62—C63	−1.7 (6)
Br42—C42—C43—C44	−179.2 (3)	C67—C61—C62—C63	177.0 (3)
C42—C43—C44—C43^i^	−0.2 (6)	C62^i^—C61—C62—Br62	177.1 (2)
C42—C43—C44—Br44	179.4 (2)	C67—C61—C62—Br62	−4.2 (5)
C52^ii^—C51—C52—C53	0.9 (6)	C61—C62—C63—C64	0.4 (5)
C57—C51—C52—C53	−178.7 (3)	Br62—C62—C63—C64	−178.4 (3)
C52^ii^—C51—C52—Br52	−178.48 (19)	C62—C63—C64—C63^i^	1.0 (6)
C57—C51—C52—Br52	1.9 (5)	C62—C63—C64—Br64	−179.3 (2)

Symmetry codes: (i) *x*, −*y*+1/2, *z*; (ii) *x*, −*y*+3/2, *z*.

### (RNC-II) 1,3,5-Tribromo-2-isocyanobenzene - polytype II Crystal data

**Table:**

C_7_H_2_Br_3_N	*D*_x_ = 2.595 Mg m^−^^3^
*M**_r_* = 339.83	Mo *K*α radiation, λ = 0.71073 Å
Orthorhombic, *P**n**m**a*	Cell parameters from 2721 reflections
*a* = 13.5916 (18) Å	θ = 3.0–27.4°
*b* = 10.1464 (13) Å	µ = 13.84 mm^−^^1^
*c* = 12.6158 (16) Å	*T* = 173 K
*V* = 1739.8 (4) Å^3^	Block, colourless
*Z* = 8	0.40 × 0.35 × 0.20 mm
*F*(000) = 1248	

### (RNC-II) 1,3,5-Tribromo-2-isocyanobenzene - polytype II Data collection

**Table:**

Bruker APEXII CCD diffractometer	1638 reflections with *I* > 2σ(*I*)
Radiation source: sealed tube	*R*_int_ = 0.078
φ and ω scans	θ_max_ = 27.5°, θ_min_ = 2.2°
Absorption correction: multi-scan (*SADABS*; Bruker, 2002)	*h* = −17→17
*T*_min_ = 0.170, *T*_max_ = 0.333	*k* = −13→13
19459 measured reflections	*l* = −16→16
2105 independent reflections	

### (RNC-II) 1,3,5-Tribromo-2-isocyanobenzene - polytype II Refinement

**Table:**

Refinement on *F*^2^	Hydrogen site location: inferred from neighbouring sites
Least-squares matrix: full	H-atom parameters constrained
*R*[*F*^2^ > 2σ(*F*^2^)] = 0.025	*w* = 1/[σ^2^(*F*_o_^2^) + (0.0121*P*)^2^ + 1.0004*P*] where *P* = (*F*_o_^2^ + 2*F*_c_^2^)/3
*wR*(*F*^2^) = 0.055	(Δ/σ)_max_ = 0.001
*S* = 1.06	Δρ_max_ = 0.44 e Å^−^^3^
2105 reflections	Δρ_min_ = −0.48 e Å^−^^3^
116 parameters	Extinction correction: *SHELXL*, Fc^*^=kFc[1+0.001xFc^2^λ^3^/sin(2θ)]^-1/4^
0 restraints	Extinction coefficient: 0.00269 (12)

### (RNC-II) 1,3,5-Tribromo-2-isocyanobenzene - polytype II Special details

**Table:**

Geometry. All esds (except the esd in the dihedral angle between two l.s. planes) are estimated using the full covariance matrix. The cell esds are taken into account individually in the estimation of esds in distances, angles and torsion angles; correlations between esds in cell parameters are only used when they are defined by crystal symmetry. An approximate (isotropic) treatment of cell esds is used for estimating esds involving l.s. planes.

### (RNC-II) 1,3,5-Tribromo-2-isocyanobenzene - polytype II Fractional atomic coordinates and isotropic or equivalent isotropic displacement parameters (Å^2^)

**Table:**

	*x*	*y*	*z*	*U*_iso_*/*U*_eq_	
C121	0.3678 (3)	0.7500	0.5680 (3)	0.0162 (9)	
C122	0.3637 (2)	0.6315 (3)	0.6238 (2)	0.0176 (7)	
C123	0.3573 (2)	0.6306 (3)	0.7332 (2)	0.0182 (7)	
H123	0.3541	0.5499	0.7712	0.022*	
C124	0.3559 (3)	0.7500	0.7858 (4)	0.0173 (9)	
N127	0.3793 (3)	0.7500	0.4583 (3)	0.0215 (9)	
C127	0.3909 (4)	0.7500	0.3682 (4)	0.0285 (12)	
Br122	0.36763 (3)	0.47074 (3)	0.54952 (3)	0.02456 (11)	
Br124	0.35282 (4)	0.7500	0.93610 (4)	0.02254 (13)	
C131	0.3904 (3)	0.2500	0.1747 (3)	0.0161 (10)	
C132	0.3885 (2)	0.3685 (3)	0.1192 (3)	0.0169 (7)	
C133	0.3821 (2)	0.3691 (3)	0.0100 (2)	0.0179 (7)	
H133	0.3806	0.4499	−0.0281	0.021*	
C134	0.3781 (3)	0.2500	−0.0428 (4)	0.0190 (10)	
N137	0.3955 (3)	0.2500	0.2840 (3)	0.0180 (8)	
C137	0.3995 (3)	0.2500	0.3761 (4)	0.0246 (11)	
Br132	0.39399 (3)	0.52885 (3)	0.19404 (3)	0.02480 (11)	
Br134	0.36801 (4)	0.2500	−0.19267 (4)	0.02564 (14)	

### (RNC-II) 1,3,5-Tribromo-2-isocyanobenzene - polytype II Atomic displacement parameters (Å^2^)

**Table:**

	*U*^11^	*U*^22^	*U*^33^	*U*^12^	*U*^13^	*U*^23^
C121	0.013 (2)	0.019 (2)	0.016 (2)	0.000	0.0002 (19)	0.000
C122	0.0164 (16)	0.0157 (16)	0.0207 (17)	0.0016 (14)	−0.0016 (14)	−0.0031 (13)
C123	0.0191 (17)	0.0152 (17)	0.0202 (17)	0.0013 (14)	−0.0017 (14)	0.0039 (13)
C124	0.017 (2)	0.017 (2)	0.018 (2)	0.000	0.0003 (19)	0.000
N127	0.026 (2)	0.018 (2)	0.021 (2)	0.000	0.0008 (17)	0.000
C127	0.035 (3)	0.025 (3)	0.026 (3)	0.000	0.002 (2)	0.000
Br122	0.0339 (2)	0.01579 (18)	0.0239 (2)	−0.00052 (15)	0.00210 (15)	−0.00522 (14)
Br124	0.0290 (3)	0.0231 (3)	0.0155 (2)	0.000	−0.0005 (2)	0.000
C131	0.014 (2)	0.019 (2)	0.015 (2)	0.000	−0.0026 (17)	0.000
C132	0.0158 (16)	0.0138 (16)	0.0210 (17)	0.0001 (13)	0.0003 (13)	−0.0042 (13)
C133	0.0229 (18)	0.0132 (17)	0.0174 (17)	0.0014 (14)	−0.0001 (13)	0.0035 (13)
C134	0.018 (2)	0.024 (3)	0.015 (2)	0.000	−0.0016 (18)	0.000
N137	0.019 (2)	0.017 (2)	0.018 (2)	0.000	0.0008 (16)	0.000
C137	0.024 (3)	0.019 (3)	0.030 (3)	0.000	−0.001 (2)	0.000
Br132	0.0360 (2)	0.01500 (19)	0.0234 (2)	−0.00111 (15)	−0.00260 (14)	−0.00427 (14)
Br134	0.0393 (3)	0.0222 (3)	0.0154 (3)	0.000	0.0003 (2)	0.000

### (RNC-II) 1,3,5-Tribromo-2-isocyanobenzene - polytype II Geometric parameters (Å, º)

**Table:**

C121—N127	1.393 (6)	C131—N137	1.380 (6)
C121—C122^i^	1.395 (4)	C131—C132^ii^	1.392 (4)
C122—C123	1.382 (4)	C132—C133	1.380 (4)
C122—Br122	1.882 (3)	C132—Br132	1.883 (3)
C123—C124^i^	1.381 (4)	C133—C134^ii^	1.381 (4)
C123—H123	0.9500	C133—H133	0.9500
C124—Br124	1.897 (5)	C134—Br134	1.895 (4)
N127—C127	1.147 (6)	N137—C137	1.164 (6)
			
N127—C121—C122^i^	120.4 (2)	N137—C131—C132^ii^	120.3 (2)
C122—C121—C122^i^	119.1 (4)	C132^ii^—C131—C132	119.5 (4)
C123—C122—C121	120.8 (3)	C133—C132—C131	120.5 (3)
C123—C122—Br122	119.6 (2)	C133—C132—Br132	119.9 (2)
C121—C122—Br122	119.6 (2)	C131—C132—Br132	119.5 (2)
C124—C123—C122	118.3 (3)	C132—C133—C134	118.7 (3)
C124—C123—H123	120.8	C132—C133—H133	120.7
C122—C123—H123	120.8	C134—C133—H133	120.7
C123—C124—C123^i^	122.5 (4)	C133^ii^—C134—C133	122.1 (4)
C123^i^—C124—Br124	118.7 (2)	C133^ii^—C134—Br134	118.9 (2)
C127—N127—C121	178.5 (5)	C137—N137—C131	179.8 (4)
			
N127—C121—C122—C123	176.7 (3)	N137—C131—C132—C133	−179.2 (3)
C122^i^—C121—C122—C123	−1.0 (6)	C132^ii^—C131—C132—C133	1.6 (6)
N127—C121—C122—Br122	−3.0 (5)	N137—C131—C132—Br132	0.6 (5)
C122^i^—C121—C122—Br122	179.30 (19)	C132^ii^—C131—C132—Br132	−178.6 (2)
C121—C122—C123—C124	−0.5 (5)	C131—C132—C133—C134	−0.2 (5)
Br122—C122—C123—C124	179.2 (3)	Br132—C132—C133—C134	−179.9 (3)
C122—C123—C124—C123^i^	2.1 (7)	C132—C133—C134—C133^ii^	−1.3 (7)
C122—C123—C124—Br124	−177.4 (2)	C132—C133—C134—Br134	179.3 (3)

Symmetry codes: (i) *x*, −*y*+3/2, *z*; (ii) *x*, −*y*+1/2, *z*.

### (RNC-II) 1,3,5-Tribromo-2-isocyanobenzene - polytype II Hydrogen-bond geometry (Å, º)

**Table:**

*D*—H···*A*	*D*—H	H···*A*	*D*···*A*	*D*—H···*A*
C123—H123···Br134^iii^	0.95	3.08	3.976 (3)	157
C133—H133···Br124^iv^	0.95	3.10	3.995 (3)	157

Symmetry codes: (iii) *x*, *y*, *z*+1; (iv) *x*, *y*, *z*−1.
